# Peer-Education Intervention to Reduce Injection Risk Behaviors Benefits High-Risk Young Injection Drug Users: A Latent Transition Analysis of the CIDUS 3/DUIT Study

**DOI:** 10.1007/s10461-012-0373-0

**Published:** 2012-11-11

**Authors:** Mary E. Mackesy-Amiti, Lorna Finnegan, Lawrence J. Ouellet, Elizabeth T. Golub, Holly Hagan, Sharon M. Hudson, Mary H. Latka, Richard S. Garfein

**Affiliations:** 1 Division of Epidemiology and Biostatistics, School of Public Health, University of Illinois at Chicago, Chicago, IL USA; 2 Department of Health Systems Science, College of Nursing, University of Illinois at Chicago, Chicago, IL USA; 3 Bloomberg School of Public Health, Johns Hopkins University, Baltimore, MD USA; 4 Center for Drug Use and HIV Research, National Development and Research Institutes, New York, NY USA; 5 Health Research Association, Los Angeles, CA USA; 6 The Aurum Institute, Johannesburg, South Africa; 7 Division of Global Public Health, School of Medicine, University of California, San Diego, CA USA; 8 Community Outreach Intervention Projects, School of Public Health, MC 923, University of Illinois at Chicago, 1603 W. Taylor St., Chicago, IL 60612 USA

**Keywords:** Injection drug use, Intervention, HIV, HCV, Latent class analysis

## Abstract

We analyzed data from a large randomized HIV/HCV prevention intervention trial with young injection drug users (IDUs) conducted in five U.S. cities. The trial compared a peer education intervention (PEI) with a time-matched, attention control group. Applying categorical latent variable analysis (mixture modeling) to baseline injection risk behavior data, we identified four distinct classes of injection-related HIV/HCV risk: low risk, non-syringe equipment-sharing, moderate-risk syringe-sharing, and high-risk syringe-sharing. The trial participation rate did not vary across classes. We conducted a latent transition analysis using trial baseline and 6-month follow-up data, to test the effect of the intervention on transitions to the low-risk class at follow-up. Adjusting for gender, age, and race/ethnicity, a significant intervention effect was found only for the high-risk class. Young IDU who exhibited high-risk behavior at baseline were 90 % more likely to be in the low-risk class at follow-up after the PEI intervention, compared to the control group.

## Introduction

Injection drug use is a serious public health problem in the U.S. and globally, leading to blood-borne infections including HBV, HCV, and HIV. Although the number of HIV/AIDS cases in the U.S. due to injection drug use has declined significantly since its peak in 1993, drug injection remains a major risk factor [1]. In 2007, an estimated 15 % of reported HIV cases among adults and adolescents in the U.S. (*N* = 9,200) were associated with injection drug use or sexual contact with injection drug users (IDUs). In addition, 21 % of reported HIV cases among children (<13 years) were associated with injection drug use by the mother or the mother’s sexual partner [2].

Drug injection is also responsible for most HCV transmission in the U.S., and is a major contributor to HBV transmission [3–5]. Recent surveys suggest that approximately one-third of young (18–30 years old) IDUs in the U.S. are HCV-infected, although prevalence varies widely [6]. The sharing of contaminated syringes and other injection equipment is the principal means by which drug injection exposes users to these viruses [7–11].

Despite significant advances in the prevention of HIV/AIDS and hepatitis, injection drug use continues to contribute to new infections both directly through the sharing of injection equipment, and indirectly through sexual transmission from IDUs to non-IDU sex partners [12–15]. Syringe access, opioid substitution therapy, street outreach, and counseling and testing (C&T) have been identified as important components in advancing HIV/HCV prevention [16–22], while enhanced behavioral interventions with IDUs have shown only modest effects on reducing receptive injection risk behaviors over and above standard services (i.e. risk assessment, C&T) [23–25]. However, peer education initiatives have demonstrated some success in influencing the behavior of IDUs trained to be peer educators [26–29]. Spanning five cities, the Third Collaborative Injection Drug Users Study (CIDUS 3) drug users intervention trial (DUIT), conducted from 2002 through 2005, is the largest randomized HIV prevention intervention trial with young IDUs in the U.S. to date. This study compared a peer education intervention (PEI) with a time-matched, attention control group receiving standard counseling and testing.

While the DUIT enhanced intervention demonstrated an overall greater decrease in injection-related HIV risk behavior compared to the control [30, 31], not all DUIT participants reduced their risk behavior. The average effect conceals a heterogeneous mix of intervention responders and non-responders. The key to improving interventions with IDUs may lie in understanding variations in responses to these interventions. Conventional approaches to the analysis of intervention effects are based on fitting a regression model for the average response pattern. However, when a behavior is highly heterogeneous, it is often useful to characterize that heterogeneity in order to identify discrete patterns of change. If we can identify certain behavioral profiles that are associated with intervention success, future efforts with that intervention can be targeted to individuals with that particular profile.

Latent variable mixture models are used to capture this heterogeneity [32]. Latent class analysis is a person-centered method for empirically identifying distinct patterns or subtypes, based on a set of observed categorical variables [33]. Latent transition analysis is an extension of the latent class model that is used to examine transitions across classes over time [34, 35]. By incorporating an intervention effect in a latent transition model, we can test whether the intervention effect is consistent or varies across classes [36]. In this study, we used latent variable mixture modeling to: (1) identify distinct classes of individuals with unique, class-specific patterns of injection risk behavior, and (2) test variation in the effectiveness of the intervention across these risk classes.

## Methods

### Study Design

We analyzed existing CIDUS3 DUIT data collected between May 2002 and January 2004 from 1569 eligible participants who were recruited in five US cities: Baltimore MD, Chicago IL, Los Angeles CA, New York City NY, and Seattle WA. Details of the study objectives, design and methodology have been described elsewhere [37, 38]. Participants were eligible for the trial if they reported injecting illicit drugs in the past 6 months, intended to reside in their recruitment city for at least the next 12 months, spoke English, were between 15 and 30 years old, and tested antibody-negative for HIV and HCV at baseline. Eligible participants who attended the post-test counseling session where they were invited to participate in the trial were included in the baseline analysis.

Individuals who consented to participate in the trial (*N* = 854) were randomly assigned to either the PEI, or a video-discussion control group. Participants in both conditions attended six group sessions over a 3-week period. All participants attended at least the first session; attendance at each of the remaining sessions was reasonably high and similar across trial arms (average 77 % for PEI, 78 % for control). Participants were compensated for time and travel after each visit, according to local guidelines—$20–$40 for ACASI interviews, $10–15 for each test result visit, and $20–25 for each intervention session attended (with four sites offering a $40 bonus for attending all six sessions).

PEI participants were informed that the purpose of the intervention was to train them to be peer educators who could help in the fight against AIDS and hepatitis in their communities. Talking to others about HIV and HCV prevention, in a pro-social role of peer educator, was expected to motivate behavior change in the educators [38]. In the first four sessions, participants learned what it meant to be a peer educator and were given tools appropriate to this role. The first two sessions focused on injection-related risk and the third and fourth sessions focused on sexual risk behavior. The format included videos; interactive discussions; exercises in skills building, role playing, and practice; and other factors such as offering community resources, information, and tools (e.g., condoms) at every session. In the fifth session, participants were given an opportunity to practice sharing risk-reduction information in a community setting, for example, by engaging in supervised peer outreach or staffing an information table at a community center or health fair. These experiences were followed by debriefing and feedback from the intervention facilitator. The sixth session consisted of a group debriefing about the community-based peer education session, followed by a goal-setting activity.

The control condition consisted of watching videos followed by facilitated discussion for an equivalent amount of time as the PEI sessions. Videos addressing social and health issues were chosen to be of interest to the target population, yet devoid of specific HIV/HCV risk-reduction content.

At baseline and follow-up visits, participants completed a behavioral assessment using audio computer-assisted self-interview (ACASI) technology to minimize socially desirable responding. Retention rates for the 3- and 6-month follow-up visits were 64–76 %, respectively, with 83 % of the sample (*N* = 712) completing at least one follow-up interview. Institutional review boards at the CDC and all collaborating institutions approved the study protocol, and all individuals provided written, informed consent to participate in the study.

### Measures

#### Sociodemographic Measures

Respondents provided information on sociodemographic characteristics, including sex, age, race/ethnicity, homelessness, incarceration, and sources of income (legal and illegal).

#### Injection Drug Use

Respondents were asked about the types of drugs they injected, how often they injected, how often they injected with other IDUs, how many different people they injected with, and their relationship to the people they injected with.

#### Injection-Related HIV/HCV Risk

Seven measures of injection risk behavior were included in this analysis: relative frequency of sharing syringes, cookers, cotton filters, and rinse water, frequency of using a new sterile syringe to divide drugs, frequency of cleaning needles with bleach, and the number of people sharing a syringe with the respondent. Relative frequency of sharing syringes and other injection equipment, using new sterile syringes to divide drugs, and cleaning needles with bleach were measured on a 7-point Likert-type scale, labeled from “always” to “never,” with “about half the time” as the midpoint. Measures of “safe” behavior (using new sterile syringes to divide drugs, and cleaning syringes with bleach) were reversed so that for all measures higher scores represented more risky behavior. Since the distributions of these variables were highly skewed, they were re-coded into three categories: never, less than half the time, and half the time or more. The number of injection partners sharing a syringe with the respondent was also recoded into three categories: none, one, and more than one.

### Analysis

#### Baseline

The seven categorical measures of injection risk behavior were used to identify distinct classes of risk behavior. Using the baseline data from all trial-eligible respondents (*N* = 1569), we conducted latent class analyses using Mplus version 6.1 [39]. We fit models with two to six classes and compared the log-likelihood and goodness-of-fit indices. This first step helps to reveal how many classes fit the data best.

After selecting the best-fitting latent class model, the class probabilities and most likely class patterns were further analyzed using Stata version 11. Multinomial logistic regressions were conducted predicting most likely class membership from sociodemographic variables, injection frequency, and HIV/HCV risk knowledge and attitudes. These analyses provide information about how the classes differed at baseline, that is, what characteristics are associated with each class.

As a test of selection bias, the distribution of classes was compared among participants who were randomized to a trial condition, and those who were not randomized (i.e. they either did not consent to trial participation or failed to show up). This comparison provides information on whether certain classes of participants were more or less likely to participate in the trial.

#### DUIT Trial

The analysis of the DUIT trial used data from 708 participants who completed at least one follow-up interview and had non-missing injection risk data. The purpose of this analysis was to test whether the intervention was equally effective across classes, or whether some classes were more responsive than others. First, latent class models with three to five classes were fit separately for each time point to verify that the number of classes selected based on the baseline analysis was appropriate at all points. Then latent transition models were conducted for baseline to three-month, and baseline to 6-month follow-up data. Models with thresholds constrained to be equal over time were compared with models allowing thresholds to vary, using the Satorra-Bentler χ^2^ difference test based on log-likelihood values and scaling correction factors obtained with the MLR estimator in Mplus [40]; see http://www.statmodel.com/chidiff.shtml. Finally, the intervention effect was added to the model as a known class, and a model with thresholds constrained across trial arms was compared with an unconstrained model. To test for differential response across classes, the probability of low-risk class membership at follow-up was analyzed in Stata 11 using a generalized linear model with a logit transformation, with intervention arm and the most likely class at baseline, and their interaction, as predictors. Marginal effects were computed for intervention arm within each baseline class.

## Results

### Sample Demographics

The baseline sample was 33 % female, with a mean age of 23.5 (range 15–30; 38 minors age 15–17). Two-thirds of respondents were non-Hispanic White, 18 % were Hispanic, 11 % were non-Hispanic Black, and 6 % were other race/ethnicity. Forty-seven percent reported being homeless, and 18 % reported being in jail or prison in the past 6 months.

### Baseline

**Fig. 1:**
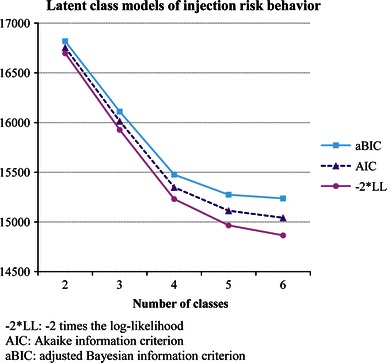
Goodness of fit measures for latent class models

Based on model fit indices and log-likelihood change (see Fig. 1), the four-class model was clearly better than the three-class model, while the five-class model resulted in a relatively small improvement over the four-class model. Although the bootstrap likelihood ratio test indicated that the five-class model resulted in a significant improvement in fit (*p* < 0.0001), the additional class extracted comprised <10 % of the sample and we were not convinced that it contributed substantively to the model. The four-class model had very good classification quality (Entropy = 0.899), and the average latent class probabilities for most likely latent class membership ranged from 0.926 to 0.966. Based on the fit indices as well as conceptual considerations, we proceeded with the four-class model.

**Fig. 2:**
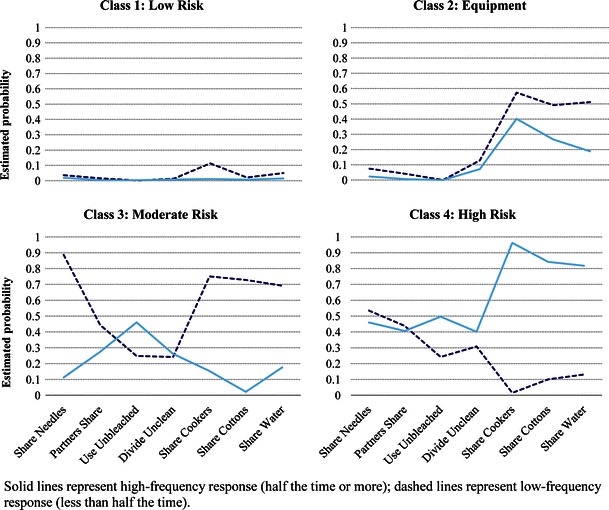
Four latent classes of injection risk behavior

Figure 2 shows the unique patterns of injection risk behaviors for each class in the four class model: (1) overall low risk (33 %), (2) equipment sharing (22 %), (3) moderate risk characterized by low-frequency sharing of syringes (19 %), and (4) overall high risk (27 %). For each class, the *x*-axis includes the seven risk behaviors, and the *y*-axis represents the probabilities of high (solid lines) and low frequency (dashed lines) responses for each risk behavior. The five-class model (not shown) split the equipment sharing class into two classes, one that shared equipment often, and one that shared infrequently.

The low-risk class exhibited very little risk behavior; about 11 % of individuals in this class shared cookers less than half the time. In the equipment-sharing class, 40 % shared cookers half the time or more, while 57 % shared cookers less than half the time. Syringe sharing was very unlikely in this group, and no one in this group injected with a used syringe without cleaning it with bleach. Everyone in the moderate risk class shared syringes, most (89 %) less than half the time; however only 29 % always cleaned their shared syringes with bleach. The high-risk class was characterized by high frequency of sharing equipment, and low or high frequency of syringe sharing with a majority not cleaning shared syringes with bleach.

Table 1 shows some demographic and injection-related characteristics of the four injection risk classes. The high-risk class had the highest percentage of women, the lowest mean age, the highest rates of homelessness and income from illicit activities. The classes did not differ significantly on rates of incarceration. The low-risk class was distinguished by the highest percentages of Hispanic and Black IDUs and the fewest injection partners. Both the low-risk and equipment-only classes had lower frequencies of injections compared to the moderate and high-risk classes.

**Table 1:** Baseline characteristics of four latent classes of injection risk behavior

	Injection risk behavior latent class				LR χ^2^	*p*	*N*
	Low risk (*N* = 519)	Equip (*N* = 336)	Mod risk (*N* = 289)	High risk (*N* = 416)			
*Age*							
Mean	23.92	23.67	23.35	22.92	19.90	0.0002	1560
Std Err	0.16	0.19	0.21	0.17			
*Gender (%)*							
Male	74.18	64.29	70.59	58.17	29.56	<0.0001	1560
Female	25.82	35.71	29.41	41.83			
*Race/ethnicity (%)*							
White	58.57	68.45	69.55	70.67	25.64	0.0023	1560
Black	14.64	10.42	9.00	7.21			
Hispanic	20.81	16.67	14.88	17.07			
Other	5.97	4.46	6.57	5.05			
Homeless past 6 months (%)	41.39	50.15	46.37	51.44	11.23	0.0106	1557
Slept in non-dwelling (%)^a^	33.08	39.29	36.81	46.39	17.70	0.0005	1557
Ever incarcerated (%)	73.03	66.37	70.59	65.87	7.29	0.0633	1560
Incarcerated past 6 months (%)	16.54	16.87	19.01	19.32	1.70	0.6380	1544
Job income (%)	57.23	68.45	62.98	60.82	11.33	0.0101	1560
Illicit activity income (%)	37.76	44.64	48.10	55.05	28.74	<0.0001	1560
*Times injected* ^*b*^							
Mean	165.34	186.69	224.46	250.84	45.96	<0.0001	1542
Std Err	8.22	8.79	13.11	11.78			
*People injected with* ^*b*^							
Mean	2.69	4.45	5.28	5.52	72.57	<0.0001	1530
Std Err	0.28	0.30	0.44	0.34			
*Times injected with others* ^*b*^							
Mean	43.67	80.14	101.69	150.83	124.41	<0.0001	1435
Std Err	4.59	6.63	10.93	10.29			

^a^In the past 6 months, have you slept in a car, abandoned building, public park, shelter, squatting place, or other non-dwelling for more than 7 nights in a row?

^b^Past 3 months

LR χ^2^ = Likelihood ratio χ^2^ from multinomial logistic regression

Table 2 shows HIV/HCV risk knowledge and attitudes by injection risk class. The four classes had similar levels of risk knowledge, but varied in risk perceptions, peer norms, and self-efficacy for safer injection. The low-risk class had higher levels of perceived risk than each of the other classes; peer norms for safer injection (against sharing syringes or equipment) were highest in the low risk class, and lowest in the high-risk class, with the moderate-risk and equipment-only classes in between. Self-efficacy for safer injection decreased steadily with increasing risk behavior across classes. Class frequencies varied significantly across trial sites; Baltimore had a larger proportion of participants in the high-risk class (34 %) than all other sites. Trial participation did not vary by class membership.

**Table 2:** Baseline HIV/HCV risk knowledge and attitudes by injection risk class

	Injection risk behavior latent class			
	Low risk (*N* = 513)	Equip (*N* = 331)	Mod risk (*N* = 283)	High risk (*N* = 415)
*HIV/HCV risk knowledge*				
Mean	64.25	66.03	65.69	65.50
Adj RRR^a^	1.00	1.00	1.00	1.00
95 % CI		(1.00–1.01)	(0.99–1.01)	(0.99–1.01)
HIV/HCV risk perception***				
Mean	4.24	3.92	3.93	3.80
Adj RRR	1.00^b^	0.59^c^	0.61^c^	0.51^c^
95 % CI		(0.49–0.70)	(0.51–0.74)	(0.43–0.60)
Peer norms: needles***				
Mean	0.49	0.36	0.33	0.24
Adj RRR	1.00^b^	0.73^c^	0.67^c^	0.50^d^
95 % CI		(0.57–0.93)	(0.52–0.86)	(0.39–0.64)
Peer norms: paraphernelia***				
Mean	0.42	0.20	0.24	0.09
Adj RRR	1.00^b^	0.55^c^	0.63^c^	0.41^d^
95 % CI		(0.44–0.70)	(0.50–0.81)	(0.33–0.53)
Self-efficacy safe injection***				
Mean	3.54	3.27	2.90	2.70
Adj RRR	1.00^b^	0.48^c^	0.24^d^	0.18^e^
95 % CI		(0.38–0.61)	(0.19–0.31)	(0.14–0.22)

*** Model LR χ^2^, *p* < 0.001

^a^Relative-risk ratio, adjusted for gender, age, race/ethnicity

^b,c,d,e^Estimates with different superscripts are significantly different, *p* < 0.05

### DUIT Trial

The four-class model of injection risk behavior that was fit to the baseline data also fit the 3-month and 6-month data well. For the baseline to 3-month latent transition model, a model with one class free to vary produced a significantly better fit than the full-invariance model (LR χ^2^, df 14 = 42.03, *p* < 0.001). Additionally freeing a second class resulted in a slightly better fit (LR χ^2^, df 14 = 25.41, *p* = 0.031). The baseline to 6-month latent transition model with invariant thresholds was accepted, as the non-invariance model did not result in a significantly better fit (LR χ^2^, df 54 = 62.41, *p* = 0.20). Given the variations in class structure, as well as the smaller number of participants with data at the three-month follow-up, we decided to focus the analysis on baseline and 6-month follow-up responses only. The model with thresholds invariant across trial arms was accepted based on the χ^2^ difference test (LR χ^2^, df 56 = 59.36, *p* = 0.35).

The low-risk class increased substantially at follow-up in both conditions; at baseline the probability of the low-risk class was 32 % and at follow-up it was 69 %. At the same time, the high-risk class probability decreased from 24 to 8 %, the moderate-risk class probability decreased from 21 to 10 %, and the equipment-sharing class decreased from 23 to 12 %. Latent class transition probabilities for each condition are shown in Table 3. The diagonal values include participants who remained in the same class at both time points. For example, the probability of a low-risk participant remaining in the low-risk class was 87 % in the control arm and 95 % in the PEI arm. The marginal effect for this baseline low-risk class reached significance (dy/dx = 0.069, 95 % CI 0.003–0.14; *z* = 2.04, *p* = 0.041) and remained significant when adjusted for city, age, race, and sex. The off-diagonal values represent transition across classes. For example, in the control arm, the probability of a high-risk participant transitioning to the low-risk class was 37 %, and in the PEI arm the probability was 53 %. The marginal effect of intervention arm for the baseline high-risk class was statistically significant (dy/dx = 0.157, 95 % CI 0.02–0.29; *z* = 2.27, *p* = 0.023) and remained significant when adjusted for city, age, race, and sex.

**Table 3:** Probabilities of class membership at follow-up by baseline class and intervention arm

Baseline class^a^	Control (*N* = 343)				PEI (*N* = 365)			
	Low	Equipment	Moderate	High	Low	Equipment	Moderate	High
Low	87 %	5 %	3 %	5 %	95 %	0 %	3 %	2 %
Equipment	71	18	7	4	64	26	7	3
Moderate	61	11	22	6	68	6	18	8
High	**37**	22	16	25	**53**	18	15	14

Diagonal values are percentages of participants who remained in the same class from baseline to follow-up; off-diagonal values are percentages of participants transitioning across classes

Bolded values are significantly different (*p* < 0.05)

^a^Most likely class based on posterior probabilities

Closer inspection of the most likely class patterns revealed that the difference in posterior probabilities for the baseline high-risk participants in the control versus the PEI condition was possibly due to greater loss-to-follow-up in the control condition; 13 % of high-risk participants in the control condition did not return for the 6-month follow-up, versus 4 % of those in the PEI condition (χ^2^ (1) = 4.13, *p* = 0.042). Using complete cases only, the intervention effect remained significant for baseline high-risk participants (dy/dx = 0.16, 95 % CI 0.01–0.31, *z* = 2.10, *p* = 0.036), however the effect for baseline low-risk participants did not (*p* = 0.061).

## Discussion

In this sample of young IDUs recruited in five different cities, we identified four distinct classes of injection risk behavior. One-third of the sample exhibited little or no risk behavior (low risk) at baseline. Another group was characterized by sharing mainly equipment other than syringes; participants in this class either refrained from sharing syringes, or always cleaned the syringes with bleach. The third class (moderate risk) was characterized by low frequency sharing of syringes and equipment. Participants in the high-risk group shared equipment frequently, shared syringes at least some of time, and were more likely to share syringes frequently.

The latent transition analysis indicated that the DUIT PEI intervention was most beneficial for young IDUs who exhibited high-risk behavior. The PEI was marginally more effective than the control intervention for maintaining low-risk behavior, and had no significant effect among IDUs in the moderate-risk and equipment-sharing classes. Individuals in these lower risk classes were likely to switch to low-risk behavior regardless of the intervention, with about two-thirds transitioning to the low-risk class. Theoretical explanations of risk reduction among peer interventionists have included cognitive consistency, social identity theory, and social reinforcement [27, 41–43]. Developing a pro-social identity, positive social reinforcement from community members, and cognitive dissonance associated with continued risk behavior, can influence motivation and self-efficacy for risk reduction [41]. For IDUs with high-risk behavior, the PEI may provide the social-cognitive stimulus they need to move to a higher level of motivational readiness for behavior change [44, 45]—to move from contemplation to preparation, and from preparation to action. IDUs with lower levels of risk behavior may respond just as well to less intensive interventions.

These results suggest that targeting the PEI intervention to young IDUs with a high-risk profile may be an efficient approach. One caveat however, is that intervening with a uniform group of high-risk IDUs may produce different results than with a mixed group of various risk levels. There may be some unmeasured group dynamics involved that influence the outcomes. Also, this analysis did not address the relationship between behavior change and observed HIV or HCV infection. Reductions in the use of shared syringes and other equipment should in theory lead to fewer infections, however it may be difficult to demonstrate such an effect without very large samples and sufficient follow-up time [26]. Behavioral interventions should be considered as one part of a multi-component strategy to address HIV and HCV prevention among IDUs [46].

It is important to note that young IDUs of all types were included in the trial. High-risk young IDUs were no more or less willing to participate in the intervention than their low-risk peers. However, the large proportion of low-risk participants included in the trial raises concerns. On one hand, maintaining low-risk behavior may be as important as preventing high-risk behavior. On the other hand, if individuals with low-risk behavior generally remain low-risk, the power of the study is compromised by including them in the trial. Moreover, when implementing a prevention program, focusing resources on the high-risk groups may be the most cost effective approach.

The demographic and behavioral characteristics of the risk classes are consistent with previous research [47–50]. IDUs in the high-risk class were younger, and more likely to be female, White, have unstable housing, and income from illicit activities, and injected more often compared to those in the low-risk class. The moderate-risk and equipment-only classes generally had demographic and behavior patterns in between the two extreme groups and similar to each other. However, on number of injection partners, the low-risk class differed from the other three classes which were all similar to one another. In terms of psychosocial variables, the high-risk class had the lowest levels of perceived risk, and peer norms and self-efficacy for safer injection. These constructs might be useful as proxy measures of risk behavior for selecting intervention participants.

## Conclusions

The results of this analysis indicate that the PEI had a significant impact on self-reported injection behavior among young IDUs with high-risk injection behavior. For young IDUs who are not high-risk, standard counseling and testing interventions may be as effective as enhanced interventions. Targeting the PEI to high-risk young IDUs may achieve significant behavior change at a lower cost.

## References

[1] Santibanez S, Garfein R, Swartzendruber A, Purcell D, Paxton L, Greenberg A (2006). Update and overview of practical epidemiologic aspects of HIV/AIDS among injection drug users in the United States. J Urban Health..

[2] Centers for Disease Control and Prevention. HIV/AIDS Surveillance Report, 2007. Vol. 19. Atlanta, GA: U.S. Department of Health and Human Services; 2009. Available from http://www.cdc.gov/hiv/topics/surveillance/resources/reports.

[3] Alter MJ (2007). Epidemiology of hepatitis C virus infection. World J Gastroenterol.

[4] Institute of Medicine (2010). Hepatitis and liver cancer: a national strategy for prevention and control of Hepatitis B and C.

[5] Thorpe LE, Ouellet LJ, Levy JR, Williams IT, Monterroso ER (2000). Hepatitis C virus infection: prevalence, risk factors, and prevention opportunities among young injection drug users in Chicago, 1997–1999. J Infect Dis.

[6] Amon JJ, Garfein RS, Ahdieh-Grant L, Armstrong GL, Ouellet LJ, Latka MH (2008). Prevalence of Hepatitis C virus infection among injection drug users in the United States, 1994–2004. Clin Infect Dis.

[7] Garfein RS, Vlahov D, Galai N, Doherty MC, Nelson KE (1996). Viral infections in short-term injection drug users: the prevalence of the hepatitis C, hepatitis B, human immunodeficiency, and human T-lymphotropic viruses. Am J Public Health.

[8] Hagan H, McGough JP, Thiede H, Weiss NS, Hopkins S, Alexander ER (1999). Syringe exchange and risk of infection with hepatitis B and C viruses. Am J Epidemiol.

[9] Tibbs CJ (1995). Methods of transmission of hepatitis C. J Viral Hepat..

[10] Strathdee SA, Patrick DM, Currie SL, Cornelisse PGA, Rekart ML, Montaner JSG (1997). Needle exchange is not enough: lessons from the Vancouver injecting drug use study. AIDS.

[11] Thorpe LE, Ouellet LJ, Hershow R, Bailey SL, Williams IT, Williamson J (2002). Risk of hepatitis C virus infection among young adult injection drug users who share injection equipment. Am J Epidemiol.

[12] Thiede H, Hagan H, Campbell JV, Strathdee SA, Bailey SL, Hudson SM (2007). Prevalence and correlates of indirect sharing practices among young adult injection drug users in five U.S. cities. Drug Alcohol Depend.

[13] Centers for Disease Control and Prevention (2009). HIV-associated behaviors among injecting drug users—23 cities, United States, May 2005–February 2006. Morb Mortal Wkl Rep..

[14] Bluthenthal RN, Anderson R, Flynn NM, Kral AH (2007). Higher syringe coverage is associated with lower odds of HIV risk and does not increase unsafe syringe disposal among syringe exchange program clients. Drug Alcohol Depend.

[15] Huo D, Ouellet LJ (2009). Needle exchange and sexual risk behaviors among a cohort of injection drug users in Chicago, Illinois. Sex Transm Dis..

[16] Des Jarlais DC, Perlis T, Arasteh K, Torian LV, Hagan H, Beatrice S (2005). Reductions in hepatitis C virus and HIV infections among injecting drug users in New York City, 1990–2001. AIDS.

[17] Coyle SL, Needle RH, Normand J (1998). Outreach-based HIV prevention for injecting drug users: a review of published outcome data. Public Health Rep.

[18] Wodak A, Cooney A (2006). Do needle syringe programs reduce HIV infection among injecting drug users: a comprehensive review of the international evidence. Subst Use Misuse.

[19] Gibson DR, Flynn NM, Perales D (2001). Effectiveness of syringe exchange programs in reducing HIV risk behavior and HIV seroconversion among injecting drug users. AIDS.

[20] Bluthenthal RN, Kral AH, Gee L, Erringer EA, Edlin BR (2000). The effect of syringe exchange use on high-risk injection drug users: a cohort study. AIDS.

[21] Marshall BDL, Wood E (2010). Toward a comprehensive approach to HIV prevention for people who use drugs. J Acquir Immune Defic Syndr..

[22] Sorensen JL, Copeland AL (2000). Drug abuse treatment as an HIV prevention strategy: a review. Drug Alcohol Depend.

[23] Booth RE, Kwiatkowski CF, Stephens RC (1998). Effectiveness of HIV/AIDS interventions on drug use and needle risk behaviors for out-of-treatment injection drug users. J Psychoact Drugs.

[24] Gibson DR, Lovelle-Drache J, Young M, Hudes ES, Sorensen JL (1999). Effectiveness of brief counseling in reducing HIV risk behavior in injecting drug users: final results of randomized trials of counseling with and without HIV testing. AIDS.

[25] Meader N, Ryan L, Des Jarlais DC, Pilling S. Psychosocial interventions for reducing injection and sexual risk behaviour for preventing HIV in drug users. Cochrane Database Syst Rev. 2010; (1). doi:10.1002/14651858.CD007192.pub2. 10.1002/14651858.CD007192.pub2 PMC8060015 20091623

[26] Sacks-Davis R, Horyniak D, Grebely J, Hellard M (2012). Behavioural interventions for preventing hepatitis C infection in people who inject drugs: a global systematic review. Int J Drug Policy..

[27] Latkin CA, Sherman S, Knowlton A (2003). HIV prevention among drug users: outcome of a network-oriented peer outreach intervention. Health Psychol.

[28] Latka MH, Hagan H, Kapadia F, Golub ET, Bonner S, Campbell JV (2008). A randomized intervention trial to reduce the lending of used injection equipment among injection drug users infected with hepatitis C. Am J Public Health.

[29] Tobin KE, Kuramoto SJ, Davey-Rothwell MA, Latkin CA (2011). The STEP into Action study: a peer-based, personal risk network-focused HIV prevention intervention with injection drug users in Baltimore, Maryland. Addiction.

[30] Garfein RS, Golub ET, Greenberg AE, Hagan H, Hanson DL, Hudson SM (2007). A peer-education intervention to reduce injection risk behaviors for HIV and hepatitis C virus infection in young injection drug users. AIDS.

[31] Mackesy-Amiti ME, Ouellet LJ, Golub ET, Hudson S, Hagan H, Garfein RS (2011). Predictors and correlates of reduced frequency or cessation of injection drug use during a randomized HIV prevention intervention trial. Addiction.

[32] Muthén B (1989). Latent variable modeling in heterogeneous populations. Psychometrika.

[33] McCutcheon AL (1987). Latent class analysis.

[34] Collins LM, Wugalter SE (1992). Latent class models for stage-sequential dynamic latent-variables. Multivar Behav Res.

[35] Lanza ST, Flaherty BP, Collins LM (2003). Latent class and latent transition analysis. Handbook of psychology.

[36] Collins LM, Graham JW, Rousculp SS, Fidler PL, Pan J, Hansen WB, Collins LM, Seitz LA (1994). Latent transition analysis and how it can address prevention research questions. Advances in data analysis for prevention intervention research.

[37] Garfein RS, Swartzendruber A, Ouellet LJ, Kapadia F, Hudson SM, Thiede H (2007). Methods to recruit and retain a cohort of young-adult injection drug users for the Third Collaborative Injection Drug Users Study/Drug Users Intervention Trial (CIDUS III/DUIT). Drug Alcohol Depend.

[38] Purcell DW, Garfein RS, Latka MH, Thiede H, Hudson S, Bonner S (2007). Development, description, and acceptability of a small-group, behavioral intervention to prevent HIV and hepatitis C virus infections among young adult injection drug users. Drug Alcohol Depend.

[39] MPlus [computer program]. Muthén & Muthén; Ver. 6.1, 2010.

[40] Satorra A, Bentler PM. A scaled difference Chi-square test statistic for moment structure analysis. UCLA Department of Statistics, 1999. Available from http://preprints.stat.ucla.edu.

[41] Dickson-Gomez J, Weeks MR, Convey M, Jianghong L (2011). Social psychological dynamics of enhanced HIV risk reduction among peer interventionists. J Community Psychol..

[42] Fisher JD, Misovich S, Edwards J, Tindale RS, Heath L, Posavac EJ (1990). Social influence and AIDS-preventive behavior. Social influence processes and prevention.

[43] Broadhead RS, Heckathorn DD, Weakliem DL, Anthony DL, Madray H, Mills RJ (1998). Harnessing peer networks as an instrument for AIDS prevention: results from a peer-driven intervention. Public Health Rep.

[44] Prochaska JO, DiClemente CC, Norcross JC (1992). In search of how people change: applications to addictive behaviors. Am Psychol.

[45] Prochaska JO, Redding CA, Harlow LL, Rossi JS, Velicer WF (1994). The transtheoretical model of change and HIV prevention: a review. Health Educ Behav..

[46] Degenhardt L, Mathers B, Vickerman P, Rhodes T, Latkin C, Hickman M (2010). Prevention of HIV infection for people who inject drugs: why individual, structural, and combination approaches are needed. The Lancet..

[47] Huo D, Bailey SL, Garfein RS, Ouellet LJ (2005). Changes in the sharing of drug injection equipment among street-recruited injection drug users in Chicago, Illinois, 1994–1996. Subst Use Misuse.

[48] Thorpe LE, Bailey SL, Huo D, Monterroso ER, Ouellet LJ (2001). Injection-related risk behaviors in young urban and suburban injection drug users in Chicago (1997–1999). J Acquir Immune Defic Syndr.

[49] Sherman SG, Latkin CA, Gielen AC (2001). Social factors related to syringe sharing among injecting partners: a focus on gender. Subst Use Misuse.

[50] Mandell W, Vlahov D, Latkin C, Oziemkowska M, Cohn S (1994). Correlates of needle sharing among injection-drug users. Am J Public Health.

